# Reply to ‘Challenging PD-L1 expressing cytotoxic T cells as a predictor for response to immunotherapy in melanoma’

**DOI:** 10.1038/s41467-018-05048-0

**Published:** 2018-07-26

**Authors:** Nicolas Jacquelot, Laurence Zitvogel, Alexander M. Eggermont

**Affiliations:** 1 INSERM U1015, Gustave Roussy, 114 rue Edouard Vaillant, 94805 Villejuif, France; 2 Institut de Cancérologie Gustave Roussy Cancer Campus (GRCC), 114 rue Edouard Vaillant, 94805 Villejuif, France; 3 Faculté de Médecine – Université Paris-Saclay, Kremlin Bicêtre, 94276 France; 4 CIC Biotherapie IGR Curie, CIC1428, Gustave Roussy Cancer Campus, Villejuif, 94805 France; 5 Present Address: Division of Molecular Immunology, The Walter and Eliza Hall Institute of Medical Research, 3052 Parkville, Victoria Australia

It is now widely accepted that brisk tumor immune infiltration is associated with a favorable outcome^1,2^. Melanoma is no exception to the rule^3^. Nevertheless, immune cells are often dysregulated and express a wide range of inhibitory molecules such as programmed cell death-1 (PD-1) or cytotoxic T lymphocyte antigen-4 (CTLA-4) that render them inefficient in eradicating tumors. The development of immune checkpoint inhibitors (ICI) has led to unprecedented therapeutic results in melanoma^4^ and in many other tumor types. However, the success is far from complete as many patients are or become resistant to ICI and may develop immune related adverse events under treatment^4^. Thus, a major focus of the field is now to evaluate the quality of the immune system in mapping of in details cellular composition and expression molecules in order to correlate these features with clinical outcomes. Considerable efforts have been made in finding such biomarkers associated with resistance and/or response to treatments. Their identification is of great importance as it may guide future therapeutic decisions for cancer patients

By performing a flow cytometry and ELISA-based nonbiased high content screening of blood and tumor markers involved in immune functions, on 37 stage III melanoma ex vivo exposed to ipilimumab, we discovered that PD-L1 expression by circulating CD8^+^ T cells is the best predictor of poor response to CTLA4-blockade, and confirmed this finding in a retrospective analysis of 190 stage IV metastatic melanoma patients treated with ipilimumab^5^.

In response to our work, Brochez et al.^6^, have raised a fundamental question in the evaluation and interpretation of our study whether PD-L1 expression on blood CD8^+^ T cells is a prognostic parameter of melanoma outcome or a predictive marker of response to ipilimumab treatment. Prognostic versus predictive? These two concepts are often confounded. Their identification are subject to precise statistical tests and necessitate adequate clinical trial designs for accurate interpretation^6^. Studies that evaluate a biomarker of response (predictive biomarker) require two arms, one treated and one non-treated. In this setting, significance for the interaction with treatment must be demonstrated which needs to be also controlled for other major prognostic factors of the disease^6^. One critical example is the impact of the primary tumor ulceration on melanoma outcomes and response to interferon (IFN) treatment. For instance, melanoma ulceration is very strong prognostic factor of disease outcome^8^. Interestingly, only ulcerated melanomas benefit from adjuvant IFN treatment and significance interaction with treatment has been demonstrated regardless to other prognostic factors. Thus, by definition, ulceration is also a predictive factor for IFN therapy^9,10^.

We have assessed the significance of PD-L1 expression on CD8^+^ T cells, at baseline, in several retrospective cohorts using univariate and multivariate analyses. We found a statistically significant association of its baseline expression with the overall survival of anti-CTLA-4 treated patients, which has been often referred to as a “predictive” biomarker of response to this therapy in the manuscript^5^. However, in this context, we have not evaluated its relative importance with or without ipilimumab treatment and therefore this biomarker may not be considered as a predictive biomarker. Brochez et al.^6^ is correct in pointing that out. However, we have drawn readers’ attention of the necessity of “further clinical trials” “to validate this prediction”^5^ and we did not exclude the possibility that this biomarker influences the prognosis and patients’ outcome independently of the treatment. Interestingly, PD-L1 expression is not detected on circulating CD8^+^ T cells of healthy volunteers^5^. This result strongly suggests that this parameter is likely associated with the disease and the specific immune contexture in melanoma^11^. Furthermore, we have previously published that the mean fluorescence intensity of PD-L1 on blood CD8^+^ T cells is associated with the overall survival of resected stage III melanoma patients^12^. These observations further support the results from Brochez et al.^6^ describing PD-L1 expression on circulating CD8^+^ T cells as a prognostic biomarker in melanoma.

In their correspondence, Brochez et al.^6^ also eluded to the importance of the timing of biomarker evaluation. They have also reported a positive association between PD-L1 expression with other systemic immune markers which support their first observation^13^. They notably found that PD-L1 expression is correlated with high percentage of circulating Tregs and CTLA-4 expression, an increase of myeloid derived suppressor cells (MDSC) and, a rise in indoleamine 2, 3-dioxygenase (IDO) activity. Thus, PD-L1 expression on CD8^+^ T cells is associated with a negative immune climate^13^. Interestingly, Brochez et al.^6^ reported an increase of the serum kynurenine/tryptophan (kyn/trp) ratio and suggested that this modulation was due to the functional activity of IDO. Furthermore, in non–responding melanoma patients treated with anti-CTLA-4 blockade and stereotactic body radiotherapy, an increased ratio of kyn/trp was observed during therapy, prompting for the introduction of IDO-inhibitors to reverse this climate. In this sense, they suggest that it would be of value to identify the pharmakokinetics of such markers during therapy administration. We showed that the kinetics may be of importance in Hannani et al.^14^. Soluble CD25 (sCD25) correlated with poor patient’s outcome at baseline, before ipilimumab infusion and, the less the baseline level the better. Ipilimumab injection itself induces an increase of sCD25 that reflects lymphocyte activation^14^. This illustrates clearly that the kinetics may also be crucial in evaluating the clinical relevance of a given biomarker during immunotherapy for early intervention. This point was also clearly documented by Wargo’s group in metastatic melanoma patients treated with ICI^11^.

Collectively, Brochez et al.^6^ and our group have identified the clinical relevance of PD-L1 expression on circulating cytotoxic T cells in melanoma for dictating patients’ outcome. We have described PD-L1 as a potential biomarker of resistance to CTLA-4 blockade while many other predictive candidate biomarkers of response to immune checkpoint blockers have been proposed based on retrospective analyses^15,16^. Further clinical trials are warranted to validate whether or not they are truly predictive biomarker of response to treatments, similarly to ulceration in response to IFN therapy^9^.
